# Multiple Roles of Glycans in Hematological Malignancies

**DOI:** 10.3389/fonc.2018.00364

**Published:** 2018-09-06

**Authors:** Xingchen Pang, Hongjiao Li, Feng Guan, Xiang Li

**Affiliations:** ^1^School of Biotechnology, Jiangnan University, Wuxi, China; ^2^College of Life Science, Northwest University, Xi'an, China; ^3^Wuxi Medical School, Jiangnan University, Wuxi, China

**Keywords:** glycan, hematological malignancies, N-glycosylation, O-glycosylation, glycosaminoglycan, glycosphingolipid, lectin

## Abstract

The three types of blood cells (red blood cells for carrying oxygen, white blood cells for immune protection, and platelets for wound clotting) arise from hematopoietic stem/progenitor cells in the adult bone marrow, and function in physiological regulation and communication with local microenvironments to maintain systemic homeostasis. Hematological malignancies are relatively uncommon malignant disorders derived from the two major blood cell lineages: myeloid (leukemia) and lymphoid (lymphoma). Malignant clones lose their regulatory mechanisms, resulting in production of a large number of dysfunctional cells and destruction of normal hematopoiesis. Glycans are one of the four major types of essential biological macromolecules, along with nucleic acids, proteins, and lipids. Major glycan subgroups are N-glycans, O-glycans, glycosaminoglycans, and glycosphingolipids. Aberrant expression of glycan structures, resulting from dysregulation of glycan-related genes, is associated with cancer development and progression in terms of cell signaling and communication, tumor cell dissociation and invasion, cell-matrix interactions, tumor angiogenesis, immune modulation, and metastasis formation. Aberrant glycan expression occurs in most hematological malignancies, notably acute myeloid leukemia, myeloproliferative neoplasms, and multiple myeloma, etc. Here, we review recent research advances regarding aberrant glycans, their related genes, and their roles in hematological malignancies. Our improved understanding of the mechanisms that underlie aberrant patterns of glycosylation will lead to development of novel, more effective therapeutic approaches targeted to hematological malignancies.

## Introduction

Hematological malignancies include numerous forms of acute and chronic lymphoproliferative and myeloproliferative diseases, derived respectively from the two major blood cell lineages: lymphoid and myeloid cells. Notably, lymphoma, lymphocytic leukemia, and myeloma are of lymphoid origin, whereas acute myeloid leukemia (AML), myeloproliferative neoplasm (MPN), and myelodysplastic syndrome (MDS) are of myeloid origin. A crucial factor in development of hematological malignancies is the dynamic interaction between transformed cells and the bone marrow and lymphoid tissue microenvironments. There is increasing evidence that microenvironmental changes produced by neoplastic cells progressively favor survival of these cells and determine the clinical course of disease.

**Figure 1:**
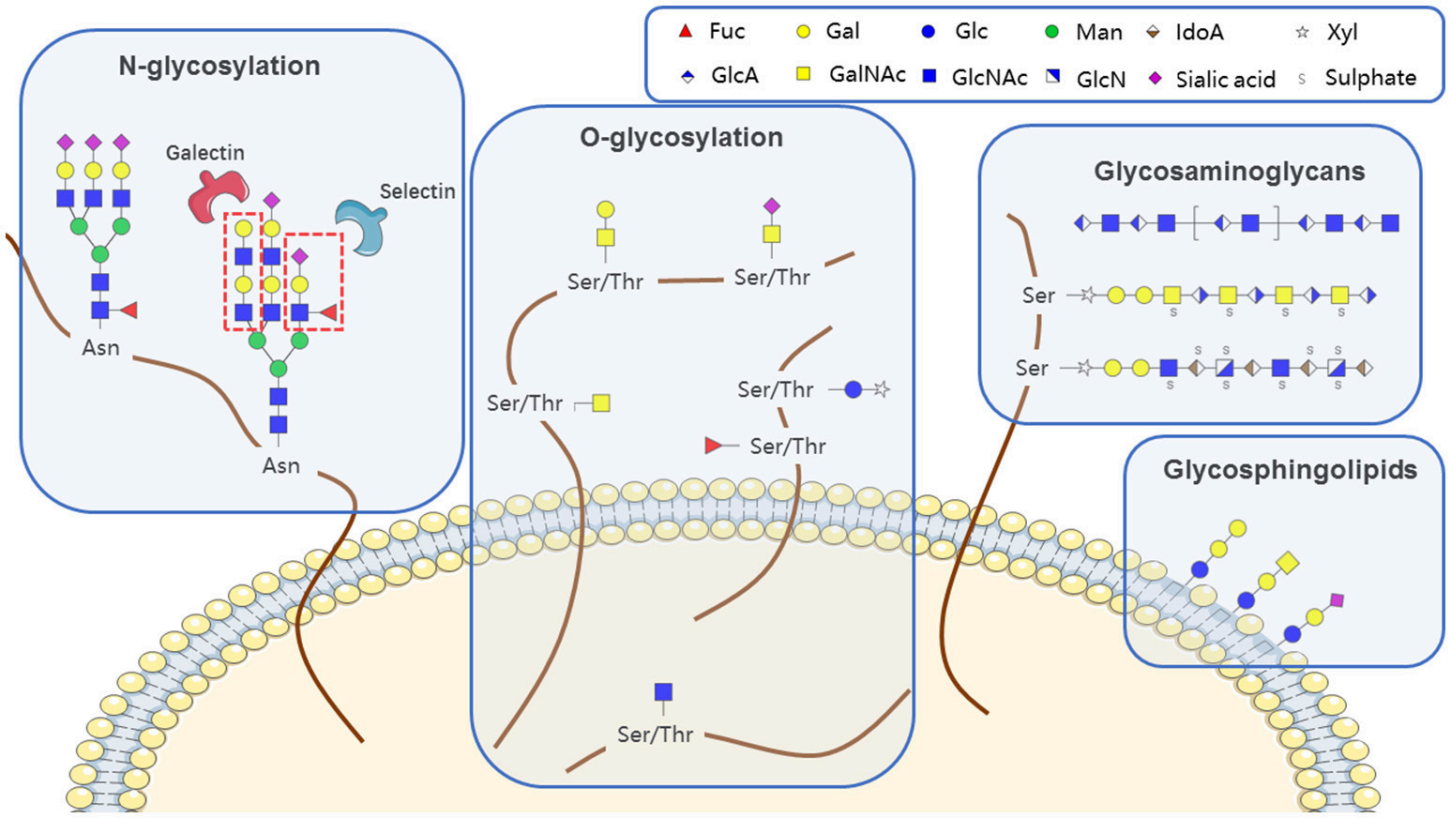
Aberrant features of glycan expression in hematological malignancies.

Glycans (polysaccharides), one of the four basic components of plant and animal cells, are the most abundant and diverse type of naturally occurring biopolymer. In addition to their well-known roles as energy sources and structural components, glycans often function as signaling effectors and cell recognition markers, typically attached to cellular proteins and lipids. Such attachment, termed glycosylation, includes N-glycosylation, O-glycosylation, glycosaminoglycans, glycosphingolipids, and glycosylphosphatidylinositol (GPI)-linked proteins, according to the conserved core structure (Figure 1). Glycans are constructed in an ordered, sequential manner that depends on distinct substrate specificities of glycosyltransferase and glycosidase enzymes. Glycosyltransferases synthesize glycan chains, whereas glycosidases hydrolyze specific glycan linkages. Glycans bind to lectins (specific carbohydrate-binding proteins) and sterically modulate molecular interactions in cells, thereby helping to control a variety of physiological mechanisms involved in health maintenance or disease development.

Aberrant glycan properties, such as hypersialylation and hyperfucosylation, are often associated with interaction of neoplastic cells with the microenvironment (1, 2). Two principal mechanisms that underlie aberrant glycan expression in hematological malignancies are incomplete synthesis and neosynthesis (3), which result mainly from altered transcription of glycosyltransferase and/or glycosidase genes.

Numerous types of glycoconjugates may potentially interfere with neoplastic cell processes or with the microenvironment, leading to malignant progression. We review here recent advances in our knowledge of aberrant glycans, and their related genes, involved in hematological malignancies. Increasingly detailed characterization of the biological functions of glycans and glycan-binding proteins (particularly galectins, selectins, and calreticulin) is progressively clarifying the physiology of hematological malignancies.

## N-glycosylation

N-glycans are linked to asparagine (Asn) residues of proteins, specifically a subset residing in the Asn-X-Ser/Thr motif and having a common, conserved five-sugar core structure. Variation in N-glycosylation of proteins, including the well-studied glycoprotein immunoglobulin G (IgG), has physiological significance in hematological malignancies. Mizuochi et al. (4) first reported truncation of N-glycan chain of IgG in a study of myeloma proteins, and many subsequent studies demonstrated effects of N-glycosylation patterns of IgGs on their biological functions. Galactose (Gal) residues are absent from total serum IgG of rheumatoid arthritis patients (5), and this altered glycosylation state activates complement via mannose-binding protein (6). Sialylation of Fc fragment core polysaccharide of IgG enhances its anti-inflammatory properties via distinct Fcγ receptors (7). Addition of fucose (Fuc) to the glycan core interferes with binding of IgG to FcγRIIIA (CD16a), and greatly reduces its capacity for antibody dependent cell-mediated cytotoxicity (ADCC) (8). Lauc et al. (9) used mass spectrometry in combination with genome sequencing to quantify N-linked IgG glycans, and identified nine genetic loci associated with IgG glycosylation, most of which showed strong associations with autoimmune disorders, inflammatory conditions, and/or hematological malignancies. Four of these were glycosyltransferase genes: ST6GAL1, B4GALT1, FUT8, and MGAT3. Leukemia cell function has been shown to be affected by aberrant N-glycosylation of other glycoproteins (e.g., CD79a, μ, CD82, CD95, MPL) besides IgG (Table 1); however, the related N-glycan synthesis genes have not been investigated in detail. Future studies utilizing an “omics” approach (combination of genomics, proteomics, and glycomics) will help elucidate glycosylation processes of single glycoproteins.

**Table 1:** Aberrant N-glycosylation of single glycoproteins associated with hematological malignancies.

**Glycoprotein**	**Disease**	**Biological impact**	**References**
IgG	Myeloma	Immunization	(4)
CD79a	B-cell chronic lymphocytic leukemia	Surface IgM expression	(10)
CD82	Acute myeloid leukemia	Trafficking and homing	(11, 12)
CD95	Myeloproliferation and B-cell lymphoma	Apoptosis regulation	(13, 14)
IgM heavy chains μ	B-cell chronic lymphocytic leukemia	Surface IgM expression	(10)
MPL	Myeloproliferative neoplasm	Cell proliferation	(15, 16)

Biosynthesis of a given N-glycan is a systemic process involving dozens of glycosyltransferases and glycosidases. Aberrant structures typically result from abnormal expression of these enzymes. The three processes most commonly involved in aberrant expression of N-glycan-related enzymes in hematological malignancies are sialylation, fucosylation, and bisecting β D N acetylglucosamine (GlcNAc), as described separately below.

### Sialylation

Sialic acids play key roles in cell recognition, adhesion, and signaling. Commonly, sialic acids are attached to galactose or N-acetylgalactosamine residues via a α2,3 or α2,6 linkage and sialic acid can be attached to another sialic acid residue via a α2,8 linkage, forming polysialic acid structure.

Enhancement of global sialylation, particularly α2,6 and α2,3 linked sialylation resulting from altered expression of the sialyltransferases ST6Gal and ST3Gal, is associated with malignancy (17). Bone marrow hematopoietic stem cells and progenitor cells form surface α2,6-linked sialic acids through the action of circulating ST6Gal1 (originating mostly from the liver), rather than endogenous ST6Gal1 (18). Elevated ST6Gal1 and ST3Gal5 mRNA levels are positively correlated with increased risk of pediatric acute leukemia. Increased expression of ST6Gal1 contributes to multidrug resistance (MDR) of leukemia cells by regulation of P-glycoprotein (P-gp) and MDR-associated protein 1 (MRP1) through phosphoinositide 3-kinase (PI3K)/Akt signaling (2). However, T cell acute lymphoblastic leukemia cell line, CCRF-CEM, decreases ST6Gal expression and, thus, α2,6 sialylation of global membrane glycoprotein to acquire resistance to microtubule targeting drug desoxyepothilone B (19). Elevated ST3Gal4 in chronic myeloid leukemia (CML) cells is associated with imatinib resistance (20). α2,3 linked sialic acids are also often found to modify O-glycans (21). Sialylation of N-glycan and O-glycan on platelets play a part in the regulation of homeostasis and clearance (22, 23). Desialylated platelets can be rapidly cleared by liver macrophages via the recognition of hepatic asialoglycoprotein receptor (also called the Ashwell–Morell receptor) (21, 24, 25).

Polysialic acid (PSA) is a linear homopolymers comprising α2,8-linked sialic acids, typically attached to neural cell adhesion molecule (NCAM). Two polysialyltransferases, ST8SiaII and ST8SiaIV, play dominant roles in PSA synthesis (26). PSA is best known for its proposed role in modulating neuronal development. However, recent researches also suggest a role for PSA in immune regulation. Drake et al. found that human NK cells modulate expression of NCAM and the degree of polymerization of its PSA according to activation state (27). They also found ST8SiaIV^−/−^ mice exhibited a specific defect in T cell development (28). Further research revealed that, Cys-Cys-chemokine receptor 7 (CCR7) and neuropilin-2 (NRP2), which are both important in immune activation, are also targets of polysialylation. After pathogen recognition, dendritic cell (DC) traffic to secondary lymphoid organs to activate naïve T cells through antigen presentation. CCR7 is the central chemokine receptor controlling DC trafficking. Polysialylation of CCR7 is essential for recognition of the CCR7 ligand CCL21. CCL21 adopts an autoinhibited conformation, which is released upon interaction with PSA (29). The interaction of the tail of CCL21 with PSA is needed for efficient ERK1/2 activation in DCs (30). NRP2 is expressed on the surface of DCs. Polysialylation of NRP2 is added to its O-Glycans, and exclusively synthesized by ST8SiaIV (31). Expression of NRP2 is up-regulated during DC maturation, coincident with increased expression of ST8SiaIV and with the appearance of PSA on the cell surface (32). NRP2 enhances the chemotactic migration of DCs toward CCL21 and promotes DC-induced activation and proliferation of T lymphocytes via the enhanced PSA-mediated effect (33, 34).

### Fucosylation

Fucosylated glycans are synthesized by a wide variety of fucosyltransferases. Fucosylation, which includes terminal fucosylation and core fucosylation, is a non-extendable modification. There are 11 different, known fucosyltransferases (FUT) that have been cloned to date. FUT1 and FUT2 are involved in the synthesis of α1,2 fucose, while FUT3, 4, 5, 6, 7, and 9 are involved in the synthesis of α1,3/α1,4 fucose, as a terminal modification that is not further elongated. FUT8 exclusively adds α1,6 fucose to the innermost GlcNAc residue of N-glycans, forming core fucosylation. However, the fucosyltransferase activity of either Fut10 or Fut11 has not been confirmed (35). Fucosylation of hematopoietic cells plays an essential functional role in homing to bone marrow, because fucosylation of cell surface molecules is responsible for binding to P- and E-selectins constitutively expressed by microvessels in marrow. Pretreatment with α1,3 fucosyltransferase VI (FucT-VI) enhances interaction of CD34^+^ stem cells and early progenitor cells with microvessels and thus promotes marrow homing during cord blood transplantation (36). Enhanced expression of sialyl Lewis X (SLe^x^) in adult T cell leukemia cells is dependent on α1,3 fucosyltransferase VII (FucT-VII) activity. Human T-lymphotropic virus 1 (HTLV 1) retrovirus, the etiologic agent of adult T cell leukemia, encodes a transcriptional activator protein (TAX) which regulates the FUT7 gene that encodes FucT-VII, the limiting enzyme controlling SLe^x^ synthesis in leukocytes (1). Overexpression of FUT8 and core fucosylation have been demonstrated in several types of cancer (37). FUT8 had an inhibitory effect on hemoglobin production and erythroid differentiation of leukemia cells during hematopoiesis (38, 39). α1,3 and α1,4 fucose does not just modify proteins but also glycosphingolipid. However, so far aberrant α1,3 and α1,4 fucosylation of glycosphingolipid in hematological malignancies has not been documented.

### Bisecting GlcNAc

The enzyme β1,4-mannosyl-glycoprotein 4-β-N-acetylglucosaminyltransferase (MGAT3) catalyzes addition of a bisecting GlcNAc residue to glycoproteins, which has a suppressive effect on several types of cancer (40). Elevation of MGAT3 activity was observed in CML patients in blast crisis and in multiple myeloma patients (41). MGAT3 overexpression in K562 leukemia cells enhanced resistance to natural killer cell cytotoxicity and spleen colonization (42), via regulation of cell recognition by bisecting GlcNAc moieties (43).

## O-glycosylation

O-glycosylation is a common covalent modification of serine (Ser) and threonine (Thr) residues of glycoproteins. The most common type of protein O-glycosylation involves α linkage via β-D-N-acetylgalactosamine (GalNAc) to the -OH of Ser or Thr by an O-glycosidic bond, resulting in “O-GalNAc glycan”; this process can be extended to various other structures. Other types of O glycans include those attached via GlcNAc, Fuc, or glucose (Glc) (44).

### O-GalNAc glycans

O-GalNAc glycans, also known as mucin-type O glycans, are found mainly in transmembrane and secreted glycoproteins, attached to certain Ser or Thr residues.

Aberrant O-GalNAc glycans, such as the disaccharide Thomsen-Friedenreich antigen (T antigen; CD176), monosaccharide GalNAc (Tn antigen; CD175), and their sialylated forms (ST antigen and STn antigen [CD175s]), result from incomplete synthesis of O-glycans and are associated with malignancy. The first step of mucin O-glycosylation, whereby Tn antigen is formed, is transfer of GalNAc from UDP-GalNAc to Ser or Thr, catalyzed by polypeptide-N-acetyl-galactosaminyltransferase (ppGalNAcT). Tn antigen is strongly expressed in chronic lymphocytic leukemia (CLL) (45), as a result of overexpression of GALNT11, a member of the ppGalNAcT family (46). Mutation or epigenetic silencing of T synthase C1GalT1 specific chaperone 1 (Cosmc; C1GALT1C1) blocked further O glycan elongation and shifted the pathway toward generation of Tn and STn through ST6GalNAc1 action (47, 48). Expression of Cosmc cDNA in Jurkat cells restored C1β3Gal-T activity and T antigen expression (49). Antitumor drugs with T or Tn antigen as therapeutic target have been developed (50, 51).

Mucin1 (MUC1; CD227) is a protein composed of ~1,000 amino acids whose molecular mass can be increased to over 1,000 kDa by addition of hundreds of O-glycans. MUC1 is translated as a single polypeptide and undergoes autoproteolysis to form two subunits: (i) N-terminal subunit (MUC1-N), containing glycosylated tandem repeats, and (ii) C-terminal transmembrane subunit (MUC1-C), which in turn forms a stable non-covalent heterodimer at the cell surface. Aberrant glycoforms of MUC1-N occurring in hematological malignancies may affect oncogenic functions of MUC1-C (52), including cell proliferation (53) and resistance to apoptosis and cytotoxic injury (54–56). MUC1 appears in a variety of hematological malignancies, including T and B cell lymphomas and myelomas (57), and is a potential prognostic marker and therapeutic target for several types of non-Hodgkin lymphoma (58, 59). MUC1 expressed in lymphoma cells is phosphorylated upon T cell receptor ligation, resulting in T cell activation (60).

Some aberrant fucosylation in O-GalNAc glycans were also observed, as referred in part 2.2.

### O-GlcNAcylation

O-GlcNAcylation refers to covalent attachment of GlcNAc sugars to Ser or Thr residues of nuclear or cytoplasmic proteins. O-GlcNAcylation, similarly to protein phosphorylation, is an enzymatic modification whose half-life is typically shorter than that of the attached protein. It is catalyzed by a single enzyme (O linked GlcNAc transferase; OGT) that transfers GlcNAc from UDP-GlcNAc to the protein substrate. N-acetyl β-glucosaminidase (OGA; also known as O-GlcNAcase), encoded by MGEA5 gene, rapidly removes the O-GlcNAc modification. These enzymes, acting together, dynamically alter the post-translational state and function of proteins in response to cellular signals. c-Myc is glycosylated by O-linked GlcNAc at Thr-58, a known phosphorylation site and “mutational hot spot” in lymphomas. Hierarchical phosphorylation of Ser-62 and Thr-58 and alternative glycosylation/ phosphorylation of Thr-58, in combination, regulate the many functions of c-Myc in cells (61). On the other hand, O-GlcNAcylation and phosphorylation may act together to trigger increased STAT5 phosphorylation levels and oncogenic transcription in hematopoietic cells (62). In CLL patients, protein O-GlcNAcylation was higher than in normal lymphocytes, and both OGT and OGA protein levels were elevated. Patients whose lymphocytes had the highest levels of O-GlcNAcylated proteins showed better prognosis, as determined by standard prognostic markers (63). The above findings indicate that leukemic lymphocytes have higher O-GlcNAcylation levels than do normal lymphocytes, with consequent blocking of signal pathways essential for rapid leukemia cell proliferation (64). OGT may also act in cooperative fashion with the tumor suppressor Ten-Eleven Translocation-2 (TET2), whose haploinsufficiency initiates myeloid and lymphoid transformation, thereby promoting post-transcriptional modification of histones and facilitating gene expression via double epigenetic modification of both DNA and histones (65, 66). OGT can induce the differentiation of MDS/AML cells *in vitro* and extend the survival rate of mice carrying leukemic cells expressing mutant ASXL1, which occurs at high frequencies in myeloid malignancies, by conjugating O-GlcNAc to ASXL1-S199 and thereby stabilizing this tumor suppressor protein (67).

### O-Fuc and O-Glc

Epidermal growth factor (EGF)-like repeats are small protein motifs (~40 amino acids) defined by six conserved cysteine residues which form three disulfide bonds. They are found in hundreds of cell surface and secreted animal proteins, and some have unusual O-linked Fuc or Glc residues. The enzymes O-fucosyltransferase 1 (POFUT1) and O-glucosyltransferase 1 (POGLUT1; hCLP46), are responsible for addition of O-Fuc and O-Glc, respectively. O-Fuc and O-Glc modifications are essential for proper Notch function. POGLUT1 is overexpressed in primary AML, T cell acute lymphoblastic leukemia (ALL), and other leukemia cell lines (68, 69). POGLUT1 overexpression enhances Notch activation and regulates cell proliferation in a cell type-dependent manner (70, 71). POFUT1 regulates lymphoid and myeloid homeostasis through modulation of Notch receptor ligand interactions (72).

## Glycosaminoglycans

Proteoglycans consist of a core protein to which one or more glycosaminoglycan (GAG) chains are covalently attached to Ser or Thr. GAGs are unbranched, often long, polysaccharides with a repeating disaccharide structure; they include heparan sulfate, chondroitin sulfate, dermatan sulfate, and keratin sulfate. The location is determined by the core protein. GAG chains, the essential functional parts, are produced by various biosynthetic pathways and are often highly sulfated, with resulting capability to bind cytokines, chemokines, or growth factors. Through such binding, they can modulate cell growth and differentiation, and thus help control embryogenesis, angiogenesis, and homeostasis. In multiple myeloma cells, the secretory-vesicle proteoglycan serglycin is the major proteoglycan expressed and constitutively secreted. High serglycin levels are present in bone marrow aspirates of >30% of newly diagnosed multiple myeloma patients, and are required for adhesion, *in vivo* growth, and vascularization of multiple myeloma cell (73, 74). Serglycin level is correlated with drug resistance in hematological malignancy cell lines (75). Serglycin is a selective marker for immature myeloid cells, and can distinguish AML from Philadelphia chromosome negative (Ph-) MPN (76). Serglycin attaches to CS4 and CS6 moieties, but not to heparin or heparan sulfate, and interacts with CD44 in a variety of hematopoietic cells (77). Cell surface proteoglycan syndecan-1 (CD138) is highly expressed in multiple myeloma cells (78) and function as a coreceptor for HGF that promotes HGF/Met signaling in MM cells (79). Heparanase is an endo-ß-d-glucuronidase that trims the heparan sulfate chains of proteoglycans, releasing biologically active fragments of heparan sulfate. Heparanase enhances shedding of syndecan-1 and high levels of heparanase and shed syndecan-1 in the tumor microenvironment are associated with elevated angiogenesis and poor prognosis in myeloma, by activating integrin and VEGF receptors on adjacent endothelial cells thereby stimulating tumor angiogenesis (80, 81). Heparan sulfate is a complex molecule because of modification by sulfation and epimerization. Heparan sulfate of syndecan-1 has more sulfated motifs than in normal plasma cells. These highly sulfated motifs bind various angiogenic and growth factors and present them to their respective receptors, and therefore play crucial roles in multiple myeloma cell survival, proliferation, and metastasis (82). A large number of transferases and modifying enzymes are involved in regulating fine structural properties of GAGs. The synthetic mechanisms responsible for generating cellular GAG structures remain poorly understood.

Hyaluronan (HA) is a unique GAG, not sulfated and not attached to a protein or lipid, that is secreted into extracellular compartments. It can interact non-covalently with various matrix components, and can be bound at the cell surface by receptors such as the adhesion molecule CD44 (83). CD44/HA interaction in monocytic cells plays crucial roles in cell migration, inflammation, and immune responses. CD44 binding ability is regulated by sialidase induced in response to cytokine stimulation (84). CD44 and HA cooperate with stromal cell-derived factor 1 (SDF-1) in trafficking of human CD34^+^ stem/ progenitor cells to bone marrow (85). A CD44 glycoform expressed on primitive CD34^+^ human hematopoietic progenitor cells is the ligand of both E-selectin (86) and L-selectin (87), through sialylated, fucosylated binding determinants on N-glycans. *Ex vivo* glycan engineering of mesenchymal stromal cell CD44, derived from adipose tissue or marrow (88), confering the native CD44 glycoform tropism to bone, which is essential for stem cell-based tissue engineering and other adoptive cellular therapies (89).

## Glycosphingolipids (GSLs)

GSLs, composed of a glycan linked to a lipid ceramide (Cer), include a series of neutral “core” structures and gangliosides, which carry one or several sialic acids. Lipid rafts on cell membranes, which contain GSLs and protein receptors, play key roles in hematological malignancies through regulation of retention, quiescence, mobilization, and homing of hematopoietic stem/progenitor cells (90). Several GSLs are markers of hematological malignancies; e.g., Gg3 in Hodgkin lymphoma (91) and Gb3 in Burkitt lymphoma (92), which result from precursor accumulation during incomplete GSL synthesis. Cer, a central molecule in sphingolipid metabolism, functions effectively as a tumor-suppressing lipid, inducing antiproliferative and apoptotic responses in a variety of cancer cells (93). Cer can be phosphorylated to form Cer-1-P, or glycosylated to form glucosylceramide (GlcCer). These derivatives have functions antagonistic to those of Cer, including anti-apoptotic effects (94). The normal balance between Cer and its derivatives is disrupted in hematological malignancies, particularly those displaying chemotherapeutic resistance (95). GlcCer was shown to be a marker for MDR cancers; it is present consistently in MDR cell lines and absent (or present at very low levels) in corresponding drug-sensitive cells (96). GlcCer synthase, the enzyme that attaches the Glc moiety to Cer, is co-overexpressed with P-glycoprotein in MDR leukemia cells (97, 98), and blocks drug-induced cell cycle arrest (99).

Gangliosides are often expressed aberrantly in malignant cells. They are important in cancer biology as both cell surface structures and molecules shed by malignant cells (100). Pediatric ALL patients showed enhanced levels of ST3GAL5 (GM3 synthase), which catalyzes α2-3-sialylation of lactosylceramide (LacCer), and resulting ganglioside GM3 (101). On the other hand, membrane type- and ganglioside-specific sialidase NEU3 was downregulated in these patients, and overexpression of NEU3 in ALL cells led to a significant increase of Cer, and induction of apoptosis in lymphoblasts (101). GM3 was found to induce monocytic differentiation in leukemia cells (102). In contrast, NEU3 inhibited megakaryocytic differentiation by degrading membrane sialic acids, maintain low level of GM3, and downregulating the PKC/ERKs/p38 MAPK pathway (103, 104).

## Lectins

Lectins are glycan-binding proteins that are typically highly selective for specific glycan structures. They include selectins, galectins, and the molecular chaperones calreticulin. Aberrant glycosylation alters the abundance of ligands for endogenous lectins, and thereby affects multiple cellular mechanisms involving the corresponding glycans.

### Selectins

SLe^x^ and SLe^a^ determinants are sialylated antigens associated with malignancy and also well-studied ligands for selectins, which are vascular cell adhesion molecules (VCAMs) belonging to the C type lectin family. There are three types of selectin: E-selectin in endothelial cells, L-selectin in lymphocytes, and P-selectin in platelets and endothelial cells. High expression of ST3 β-galactoside α2,3-sialyltransferase 6 (ST3GAL6) promotes transendothelial migration to bone marrow and survival of multiple myeloma cells, through generation of functional SLe^x^ determinants, the ligands of E-selectins on endothelial cells (105). Leukemia cells initiate interactions between Le^x^ determinants and E-selectins by directly activating resting endothelial cells. The activated endothelial cells then induce E-selectin-mediated adhesion of a subset of leukemia cells. The adherent leukemia cells are sequestered in a quiescent state and are unaffected by chemotherapy (106). Defective homing of umbilical cord blood cells to bone marrow, resulting in delayed engraftment of cord blood transplantation, is related to low fucosylation levels of cell surface molecules responsible for binding to P- and E-selectins constitutively expressed by marrow microvessels (36). L-selectin is the key factor controlling binding of B-cell CLL cells to high endothelial venule (HEV) walls of lymph nodes *in vivo*, which is related to cell growth and drug resistance of CLL (107). P-selectin glycoprotein ligand 1 (PSGL-1) is the primary ligand for L- and P-selectin, and can bind E-selectin if appropriately glycosylated (108). PSGL-1 plays a crucial role in hematogenous metastasis of lymphoid cancer cells, and led to liver and spleen colonization in *in vivo* experiments (109). The number of circulating PSGL-1-positive microparticles released from activated or apoptotic cells is a prognostic indicator for leukemia and lymphoma patients following allogeneic stem cell transplantation (110). PSGL-1 can also be a therapeutic target; immunotherapy with anti-PSGL-1 mAbs was used in combination with mCRP blockage-induced, complement-mediated lysis of multiple myeloma cells *in vivo* (111).

### Galectins

Galectins are a family of lectin molecules that display intracellular and extracellular effects making them useful agents in treatment of inflammation and tumor progression. They are classified as (i) “prototype” galectins (e.g., galectin-1 and−7) that have one carbohydrate recognition domain (CRD) and can undergo dimerization; (ii) “tandem-repeat” galectins (e.g., galectin-9) that contain two homologous CRDs in tandem in a single polypeptide chain; and (iii) galectin-3, which contains a CRD connected to a non-lectin N-terminal region responsible for oligomerization. Poly-LacNAc structure (elongated β1,4 GalT glycan chain) is a ligand for galectins. During hematopoiesis, galectin-glycan interactions maintain formation of microenvironmental niches, modulate acute and chronic inflammatory responses, and provide “on-and-off” signals that help control the balance between immune cell responsiveness and tolerance (112, 113).

The roles of galectin-1 and−3 in hematological malignancies have been well-studied. Overexpression of galectin-1 is correlated with activation of AP-1 pathway in malignant cells of Hodgkin lymphoma. Galectin-1 can serve as a predictive biomarker for relapsed or refractory Hodgkin lymphoma (114), and its serum levels reflect tumor burden and adverse clinical features (115). In clinical studies, neutralization of galectin-1 was an effective therapeutic strategy (114). In CLL, increased level of galectin-1 derived from myeloid cells was required for full stimulation of malignant cells through reduced threshold of B-cell receptor signaling (116). In multiple myeloma, galectin-1 displayed dual functions depending on CD45RA expression of malignant cells: it promoted viability and proliferation of CD45RA^−^ cells through aggregation of β1-integrin, and induced growth arrest of CD45RA^+^ cells through inhibition of ERK phosphorylation (117). Aberrant expression of galectin-1 and/or−3 in patients with MPNs is also discovered to correlate with JAK2 mutation in these diseases and state of cell differentiation (118).

Galectin-3 is overexpressed in diffuse large B-cell lymphoma, the most common type of non-Hodgkin lymphoma in adults. Cell-surface galectin-3 binds a subset of highly glycosylated CD45, the major receptor tyrosine phosphatase in B-cells, and enhances its phosphatase activity, with consequent increase of anti-apoptotic activity (119). Bone marrow stromal cells also participate in drug resistance of AML and ALL by secreting galectin-3. Soluble galectin-3 is internalized by leukemia cells and transported to the nucleus, stimulates transcription of endogenous LGALS3 mRNA and thus activates the Wnt/β-catenin signaling pathway in leukemia cells, which is critical in cytotoxic drug resistance (120, 121). In AML, high galectin-3 expression in bone marrow is an independent unfavorable prognostic factor for overall patient survival (both M3 and non-M3) (122, 123). In CML, malignant cells in chronic phase showed galectin-3 levels much higher than those in bone marrow hematopoietic cells from control subjects or acute leukemia patients. Co-culture of five CML cell lines with bone marrow stromal cells induced galectin-3 expression and increased proliferative potential and resistance to genotoxic agents (124). In multiple myeloma, the supporting effect of galectin-3 was reduced, and malignant cells were sensitized to chemotherapeutic drugs, by treatment with two agents: galectin-3 antagonist GCS-100 and an N-terminally truncated form of galectin-3 (124).

Galectin-9, in cooperation with its highly expressed ligand TIM-3, leads to T cell exhaustion and increased survival of AML cells (125). In multiple myeloma, a recombinant protease-resistant mutant form of human galectin-9 had a strong antiproliferative effect on malignant cells, dependent in part on MAPK pathway activation (126).

### Calreticulin (CALR)

Ph- MPNs are a group of diseases involving excessive bone marrow cell production, including essential thrombocytosis (ET), polycythemia vera (PV), and primary myelofibrosis (PMF). Thrombopoietin (TPO) receptor (MPL/TpoR) is the key cytokine receptor in MPN development, and activates MPL-JAK-STAT signaling in MPN stem cells (127). Incomplete MPL glycosylation was a common feature of Ph- MPNs, which was first discovered in PV patients (15). N-glycosylation on the MPL exert essential regulatory roles on MPL cell surface localization and function (128).

Calreticulin (CALR) and Calnexin(CNX), a pair of homologous C-type (calcium-dependent) lectins found in endoplasmic reticulum, act together as molecular chaperones to ensure proper folding and function of newly synthesized N-glycoproteins (129). CALR mutations are the second most frequent mutation after JAK2 in ET and PMF, nevertheless, no CALR mutations were found in patients with PV, which is specifically associated with JAK2 mutations. Moreover, CALR mutations are mutually exclusive with mutations in both JAK2 and MPL, indicating it may be responsible for the initiation of this disease (130, 131). The most frequent CALR mutations, type 1 (52-bp deletion; c.1092_1143del) and type 2 (5-bp insertion; c.1154_1155insTTGTC) accounted for 53.0% and 31.7% of all CALR mutations, respectively (130). All the mutations of CALR are insertion or deletion mutations in the last exon (exon 9) encoding the C-terminal amino acids of the protein, resulting in a novel C-terminal positively charged polypeptide tail and absence of last four amino acids (KDEL) contain the endoplasmic reticulum–retention signal (130).

CALR mutations prevent proper maturation of certain proteins (132), notably MPL in the case of Ph- MPNs. CALR mutants show alterations in MPL stability, maturation, trafficking (133), and N-glycan pattern. The majority of MPL in these mutants remains in an immature, high high-mannose form that is EndoH-sensitive (16). Pathogenic CALR mutants interacted with extracellular N-glycosylated residues of MPL via a glycan-binding site on the C-terminal tail (134, 135). Such interaction led to activation of MPL and downstream JAK2, and subsequent promotion of TPO-independent growth, accompanied by STAT5 phosphorylation. In a mouse model, engraftment of CALR mutant bone marrow cells resulted in thrombocytosis, but did not show same effect in MPL knockout mice, further confirming the MPL-dependence of calreticulin mutants (136). Moreover, CALR mutants are secreted proteins, which may be able to activate other cells, especially monocytes, to secrete inflammatory cytokines (137).

MPN patients carrying CALR mutations presented with higher platelet counts and lower hemoglobin levels than patients with mutated JAK2 (138), thus have a lower risk of thrombosis and have longer overall survival than those with a JAK2 mutation (130). CALR-positive MPNs have a more benign clinical course than the corresponding disorders associated with JAK2 or MPL mutations.

Ruxolitinib is a JAK inhibitor that ameliorates splenomegaly and constitutional symptoms associated with myelofibrosis (both PMF and post-ET/PV) (139), and is also superior to standard therapy in controlling the hematocrit, reducing the spleen volume, and improving symptoms associated with PV (140). And ruxolitinib ameliorated the thrombocytosis in CALR mutant mice and attenuated the increase in number of BM megakaryocytes and HSCs, revealing a vital role for MPL and STAT5 activation in CALR mutation-induced MPN (141).

The WHO added CALR mutations to the 2016 revision of MPN diagnostic criteria (142), while accurater and simpler prognostic models are needed to be validated for routine clinical practice (143).

## Conclusion and perspectives

Glycoconjugates are major components of animal cells and play essential roles in many physiological processes. Progress in glycobiology has led to the discovery of increasing numbers of aberrant glycans, and elucidation of the functions of certain glycans and related genes. Several serological markers currently used in the clinic are based on the detection of circulating glycoproteins or glycoconjugates with altered glycosylation. Most discoveries of aberrant glycosylation in hematological malignancy are based on genomics analysis using patient samples, mostly focusing on the abnormal expression of genes for glycosyltransferases and glycosidases. In recent years, the development of mass spectrometry-based proteomic and glycomic techniques make it possible to discover the abnormal glycoproteins and glycolipids, and cancer-related and glycosite-specific glycan structures, with the limited samples (144). Tracing back the abnormal glycan structures to the corresponding genes may sparkle this field in hematological malignancy study.

Recent studies revealed that aberrant glycosylation of hematopoietic microenvironment impact the malignant process by interacting with neoplastic cells, such as high serglycin level in multiple myeloma (74). The microenvironment of bone marrow or lymphoid tissue can be active participants to help neoplastic cells to resist drug therapy and avoid immune response. Future works are needed to understand the mechanisms of those aberrant glycan patterns not only in the aspect of cellular self-regulation, but also in the aspect of cell-cell communication within their microenvironment. The glycosylation of microenvironment could be one promising direction of hematological malignancy research in future.

The discovery of aberrant glycans and exploration of underling their mechanisms would expand their applications as diagnosis marker or therapeutic target for hematological malignancies.

For example, Roneparstat (SST0001), a 100% N-acetylated and glycol split heparin, inhibited myeloma growth and angiogenesis via disruption of the heparanase/syndecan-1 axis (145). As an heparanase inhibitor used in phase I clinical trial in hematological malignancies, Roneparstat presented an excellent safety profile, without clinically relevant systemic reactions, and an excellent tolerability profile (146). In other hands, glyco-engineered antibodies, such as anti-CD20 monoclonal antibody (Obinutuzumab [GA101]) may enhance the effective treatment for CD20^+^ B-cell non-Hodgkin's lymphoma (NHL), CD20^+^ follicular NHL, and CLL (147). *In vitro* glyco-engineering could be a powerful approach to develop monoclonal antibodies with homogenous humanized glycosylation in clinical trials of hematological malignancies.

The integrated reseach of glycomics and glycobiology with hematological malignancy will further boost the scientists to oversee the merging of biological disciplines and molecular mechanisms into physiology and disease.

## Author contributions

FG and XL conceived and designed the article frame. XP, HL, XL, and FG wrote the paper.

### Conflict of interest statement

The authors declare that the research was conducted in the absence of any commercial or financial relationships that could be construed as a potential conflict of interest. The reviewer FL and the handling Editor declared their shared affiliation.
